# Mechanical properties of epoxy nanocomposites filled with melamine functionalized molybdenum disulfide†

**DOI:** 10.1039/c8ra02689k

**Published:** 2018-06-05

**Authors:** Bin Chen, Bao-Jian Ni, Wen-Tao Liu, Qiu-Yang Ye, Si-Yuan Liu, He-Xin Zhang, Keun-Byoung Yoon

**Affiliations:** School of Materials Science and Engineering, Shenyang University of Chemical Technology Shenyang 110142 China; School of Chemistry & Chemical Engineering, Anhui University of Technology China polyhx@ciac.ac.cn; Department of Polymer Science and Engineering, Kyungpook National University South Korea kbyoon@knu.ac.kr

## Abstract

In this work, a melamine functionalized molybdenum disulfide (M-MoS_2_) was prepared and used as fillers to form epoxy (EP)/MoS_2_ nanocomposites. The effects of molybdenum disulfide (MoS_2_) and melamine functionalized molybdenum disulfide (M-MoS_2_) loading on the mechanical properties of epoxy composites were investigated and compared. With only addition of 0.8 wt% M-MoS_2_, the tensile strength and modulus of EP/M-MoS_2_ nanocomposites showed 4.5 and 4.0 times increase over the neat epoxy. Interestingly, the elongation at break value of EP was also increased with the introduction of M-MoS_2_ fillers. These properties could result from the good dispersion and strong interfacial adhesion of M-MoS_2_ fillers and the EP matrix. Therefore, this work provides a facile way to produce of high-performance EP nanocomposites.

## Abstract 2

In this work, a melamine functionalized molybdenum disulfide (M-MoS_2_) was prepared and used as fillers to form epoxy (EP)/MoS_2_ nanocomposites. The mechanical properties of EP were significantly improved, even with very low M-MoS_2_ addition.

## Graphical abstract

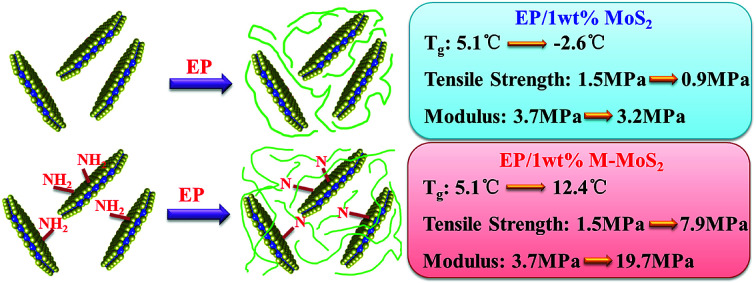

## Introduction

EP resin is an important thermoset material extensively used in a wide variety of applications such as coatings,^1^ adhesives,^2^ laminate,^3^ semiconductor encapsulate,^4^ and resin matrix composites,^5^ because of its excellent mechanical stiffness and toughness, low shrinkage, good chemical resistance and superior adhesive force to many substrates.^6–10^ Up to now, great efforts have been conducted to improve the properties of EP resin through addition of nanofillers, such as montmorillonite, polyhedral oligomeric silsesquioxanes, carbon nanotube, graphene.^11–19^

Recently, transition metal dichalcogenides (TMDCs) have attracted great interest in a wide range of research fields.^20–27^ MoS_2_ is one of the most typical TMDC.^28–31^ A monolayer of MoS_2_ reportedly has an extraordinarily high breaking strength (∼23 GPa) and Young's modulus (∼300 GPa), which are greater than those of chemically reduced graphene.^32,33^ Derived from these remarkable properties, the MoS_2_ sheets may hold considerable potential as a new EP resin reinforcement nanofiller. It has recently been reported that incorporation of MoS_2_ sheets into polymers at extraordinarily low filler content resulted in remarkable impact on the mechanical properties of the polymer, such as polystyrene, poly(methyl methacrylate), poly(vinylidene fluoride), polyvinyl alcohol, polyethylene and polypropylene.^34–39^ The resultant MoS_2_-filled polymer nanocomposites exhibited enhanced thermal stability, flame retardance and mechanical properties. With regards to EP resin, Y. Hu and Z. Gui *et al.* reported a MoS_2_-carbon nanotube reinforced EP composites.^40^ With the introduction of 2 wt% MoS_2_-carbon nanotube, the organic volatiles and carbon monoxide was suppressed, while the mechanical properties were improved. N. Koratkar *et al.* also found the addition of exfoliated MoS_2_ could enhance the mechanical properties of EP resin.^41^ Although the MoS_2_ well dispersed in the EP matrix, the interfacial adhesion between the MoS_2_ and the EP matrix are less considered.

Therefore, in this research, we report a melamine functionalized MoS_2_ and further used as fillers to reinforce the EP resin. The functionalization of MoS_2_ with melamine can prevent the agglomeration of MoS_2_, which can improve the dispersibility of MoS_2_ in EP resin. Additionally, the amine group of melamine could promote the ring-opening reaction of the EP ring and lead to form a cross-linked structure. Thus, the attached melamine enhanced the interfacial interaction between the MoS_2_ and EP matrix. Simultaneously, because the melamine and MoS_2_ were widely used flame retardant, the flame retardance of EP resin will be improved. Therefore, this work provides a facile way to produce of high-performance EP nanocomposites.

## Experimental

### Materials

Molybdenum disulfide (MoS_2_, ∼6 μm), *n*-butyllithium (2.5 M in hexane), melamine (99%), polypropylenglycol diglycidyl ether (PPGDGE, *M*_n_ ≈ 640) and polyoxypropylene diamine (D230, *M*_n_ ≈ 640) were purchased from Sigma-Aldrich and used as received. EP resin (EPON 828) was obtained from Shell.

### Preparation of organophilic MoS_2_

MoS_2_ was first exfoliated according to a literature method.^42^ Typically, 5 g MoS_2_ was placed in an autoclave, and 20 mL *n*-butyllithium (2.5 M in hexanes) was added. The autoclave was heated at 90 °C for 12 h under stirring. After that, the product was filtered and washed with anhydrous hexane (5 × 100 mL). The resultant lithium intercalated MoS_2_ was vacuum-dried and immersed in melamine aqueous solution (3 g in 1000 mL H_2_O) under ultrasonication for 4 h to produce a colloidal suspension of melamine-functionalized MoS_2_ (M-MoS_2_). The suspension was neutralized with 1 M HCl, and the products were washed with distilled water (3 × 1 L) and then washed with methanol to remove unreacted melamine. Subsequently, M-MoS_2_ was obtained by freeze-drying.

### Preparation of EP/MoS_2_ nanocomposites

The desired amount of MoS_2_ or M-MoS_2_ powder was dispersed in appropriate amount of acetone and then stirred with a magnetic stirrer at room temperature for 30 min, followed by sonicated for 60 min. After that the mixture was mixed with desired amount of EP(*m*(EPON828)/*m*(PPGDGE) = 55/45) and sonicated for another 60 min at 50 °C. The acetone was then removed by vacuum distillation under stirring with a magnetic stirrer at 80 °C. When the mixture was cooled down to 50 °C, a stoichiometric amounts of curing agent (D230) corresponding to 100% of EP resin content was added and stirred for some time. The resulting mixture was then outgassed in a vacuum oven at 60 °C for a short period of time and then cast into a Teflon mold with special size. The sample was cured at 75 °C for 2 hours and post-cured at 120 °C for 8 hours.

### Characterization of EP/MoS_2_ nanocomposites

X-ray diffraction (XRD) patterns were obtained on a Germany BRUKER D8 ADVANCE diffractometer with Cu Kα X-ray radiation. The scanning range was 2–50° with a scanning speed of 1° min^−1^. The sample specimens for transmission electron microscopy were microtomed with a diamond knife using a Leica Ultramicrotome at liquid N_2_ atmosphere. TEM images were obtained from a Japan JEOL JEM-1011 microscope operating at 200 kV in bright field mode. Differential scanning calorimeter (DSC) was conducted on Perkin-Elmer Diamond thermal analyzer. The samples were first heated from room temperature to 50 °C under N_2_ atmosphere at heating rate of 10 °C min^−1^ and was then cooled to −50 °C. Finally, the sample was reheated to 50 °C at 10 °C min^−1^. Tensile properties were performed on a USA INSTRON 5869 electronic testing instrument according to ASTM D638 at a cross-head speed of 5 mm min^−1^ at room temperature. The dumbbell like specimens (20 × 4 × 2 mm^3^) were cut from the above cured sample casted on Teflon mold. Each tensile value reported is the average of 5 tests. Optical microscope photos were obtained from an optical microscope (ANA-006, Leitz, Germany) and recorded using a charge-coupled device camera.

## Results and discussion

**Scheme 1:**
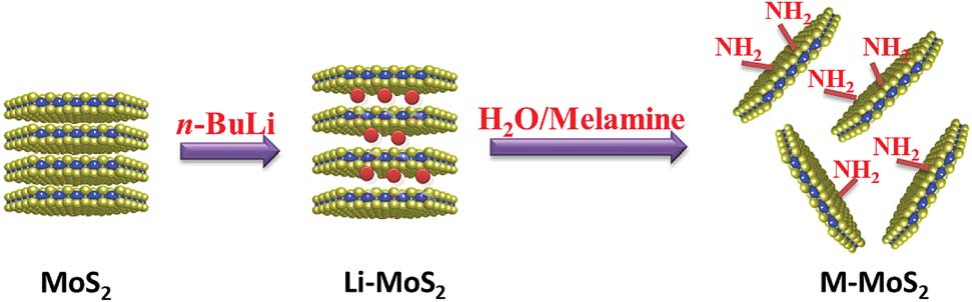
Preparation of M-MoS_2_.

**Fig. 1:**
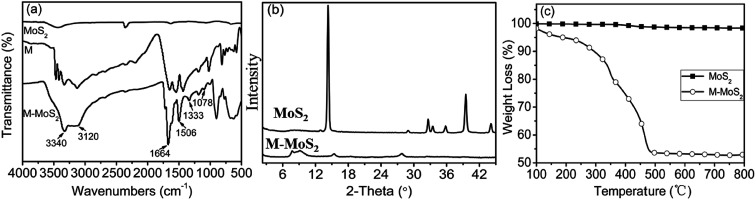
(a) FTIR spectra, (b) XRD spectra, and (c) TGA curves of MoS_2_ and M-MoS_2_.

MoS_2_ was exfoliated according to a literature method and functionalized by melamine. The exfoliation and functionalization process were given in Scheme 1. To confirm the successful functionalization of MoS_2_, Fourier transform infrared (FTIR) spectroscopy, XRD analysis and thermogravimetric analysis (TGA) were conducted. Fig. 1(a) shows the FTIR spectra of the bulk MoS_2_, melamine and M-MoS_2_. No characteristic peaks appear in the spectrum of bulk MoS_2_, whereas several sharp peaks are observed for the M-MoS_2_ sample. The two new peaks at 3340 and 3120 cm^−1^ resulting from –NH_2_ stretching of the melamine, imply the existence of the melamine on M-MoS_2_. Furthermore, the characteristic peaks of melamine (CN stretching and NH_2_ bending vibrations) at 1078 cm^−1^ and 1300–1700 cm^−1^ were apparently shifted, indicating the formation of covalent bonds between melamine and MoS_2_. These results indicate that melamine was successfully grafted onto the MoS_2_ surface.

The bulk MoS_2_ and M-MoS_2_ samples were characterized by XRD. As shown in Fig. 1(b), bulk MoS_2_ shows a single (002) diffraction peak at 2*θ* = 14.3°, which corresponds to a *d*-spacing of 0.6 nm. Upon exfoliation and functionalization, this peak becomes dramatically smaller and broader, and several new peaks are observed at lower 2*θ* values. M-MoS_2_ shows two new and very broad diffraction peaks at 2*θ* = 7.7° and 9.3° (corresponding to *d*-spacings of 1.1 and 0.9 nm, respectively), which indicate an increase in the layer distance of MoS_2_ owing to the graft of melamine. Additionally, the weak and broad diffraction peak also indicate the crystallinity of the M-MoS_2_ filler is low.

Thermal stabilities of the bulk MoS_2_ and M-MoS_2_ were investigated by TGA under nitrogen with a temperature range from the room temperature to 800 °C. As presented in Fig. 1(c), bulk MoS_2_ is clearly very thermally stable, as the mass loss is only 0.8 wt% upon heating to 800 °C. In contrast, for M-MoS_2_ sample, three degradation steps are observed. In the first step (<200 °C), the weight loss is due to the evaporation of physically adsorbed water; in the second step (220–380 °C), the weight loss is caused by decomposition of melamine that is functionalized on the MoS_2_ surface; and in the third step (>380 °C), the weight loss may be attributed to decomposition of the carbon formed on the MoS_2_ surface as a result of carbonization of melamine. The weight content of melamine in M-MoS_2_ nanofillers was calculated from the char yield of TGA measurement to ∼52 wt%. However, the content of melamine calculated from TGA is not exact, because the C_3_N_4_ will formed during the carbonization process.

**Fig. 2:**
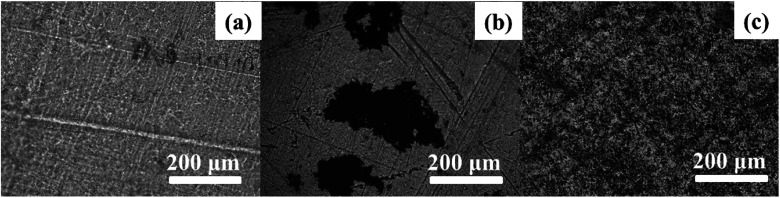
Optical micrographs of (a) EP, (b) EP/1 wt% MoS_2_ and (c) EP/1 wt% M-MoS_2_ nanocomposites.

In order to investigate the dispersion of MoS_2_ and M-MoS_2_ in the EP matrix, the resultant thin films of EP, EP/MoS_2_ and EP/M-MoS_2_ nanocomposites were prepared. The thin films were observed under an optical microscope in transparent mode; the obtained micrographs are shown in Fig. 2. It was found that the M-MoS_2_ fillers are well dispersed in the EP matrix, while MoS_2_ aggregation was observed in EP/MoS_2_ nanocomposite with 1 wt% MoS_2_ addition.

**Fig. 3:**
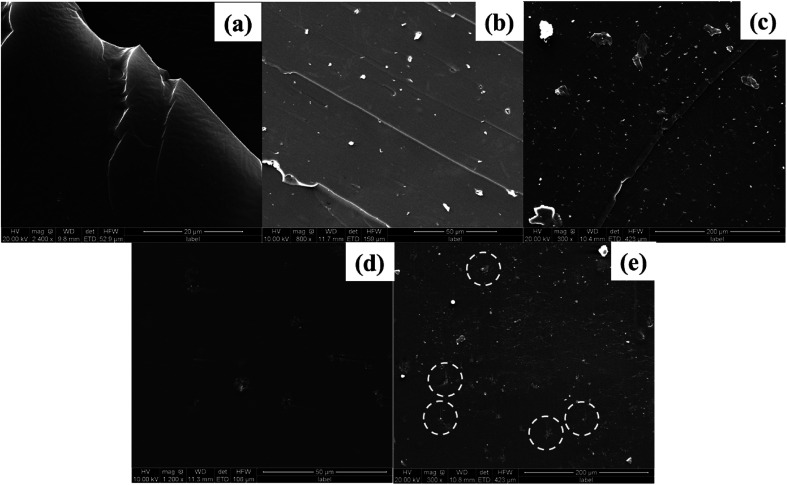
SEM images of the fractured surface of (a) EP, (b and c) EP/1 wt% MoS_2_ and (d and e) EP/1 wt% M-MoS_2_ nanocomposites.

**Fig. 4:**
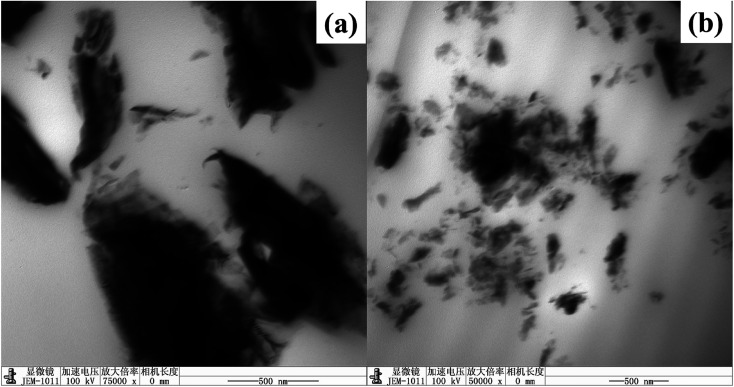
TEM images of (a) EP/MoS_2_ and (b) EP/M-MoS_2_ nanocomposite with 1 wt% filler addition.

In order to investigate the dispersion and interaction of MoS_2_ and M-MoS_2_ in the EP matrix, the resulted samples were characterized by SEM and TEM. As shown in Fig. 3, the SEM image of the EP/MoS_2_ nanocomposite exhibited a smooth fractured surface and some MoS_2_ sheet could be clear observed on the surface of the fractured surface without interaction with EP matrix. With regards to EP/M-MoS_2_ nanocomposites, the wrapped structure was observed, implying the strong interaction between M-MoS_2_ and the EP matrix. In order to fully characterized the dispersion of fillers in the nanocomposites, TEM of the microtomed section of compression molded samples was examined (Fig. 4). It was found that the M-MoS_2_ well dispersed in the EP matrix, while MoS_2_ aggregation was observed in EP/MoS_2_ nanocomposite. This morphology is correlated with the morphology obtained by optical micrographs. We therefore expected the EP/M-MoS_2_ nanocomposites will exhibit better mechanical properties than EP nanocomposite with MoS_2_ fillers.

**Fig. 5:**
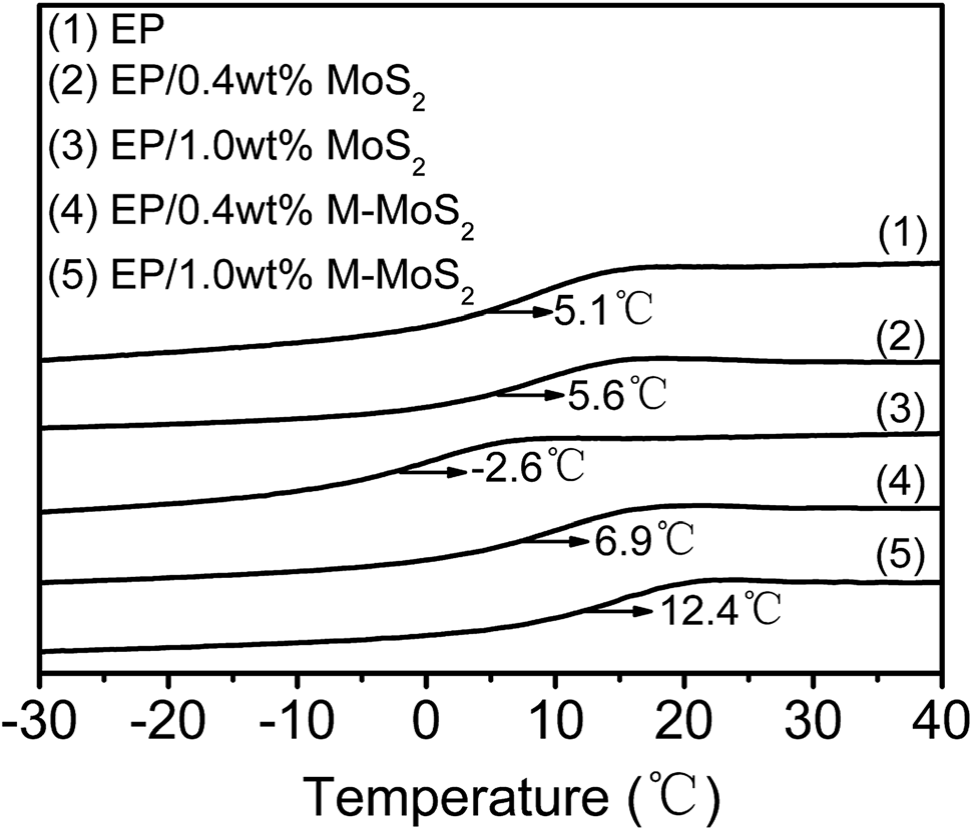
DSC curve of EP, EP/MoS_2_ and EP/M-MoS_2_ nanocomposites.

The effect of MoS_2_ and M-MoS_2_ on the glass transition temperature (*T*_g_) of EP was investigated by DSC; the typical DSC curves are shown in Fig. 5 The *T*_g_ of the virgin EP was 5.1 °C. Upon introduction of MoS_2_, the *T*_g_ value tend to decrease with increasing MoS_2_ content, while the *T*_g_ gradually increased with the M-MoS_2_ content increasing. With only 1 wt% M-MoS_2_ addition, the *T*_g_ of EP rises up to 7.3 °C. The increment of *T*_g_ refers to the reduction of matrix chain mobility by the presence of M-MoS_2_. During fabrication, melamine molecules bridged MoS_2_ with EP matrix and a strong interface was thus produced. The strong interface restricts the motion of polymer chain and thus gives rise to the increase in *T*_g_. While, with regards to EP/MoS_2_ nanocomposites, the reduced *T*_g_ are probably caused by two reasons (i) agglomeration when neat MoS_2_ fillers added. (ii) Reduction of the EP matrix's cross-linking density due to the barrier effect of MoS_2_.

**Fig. 6:**
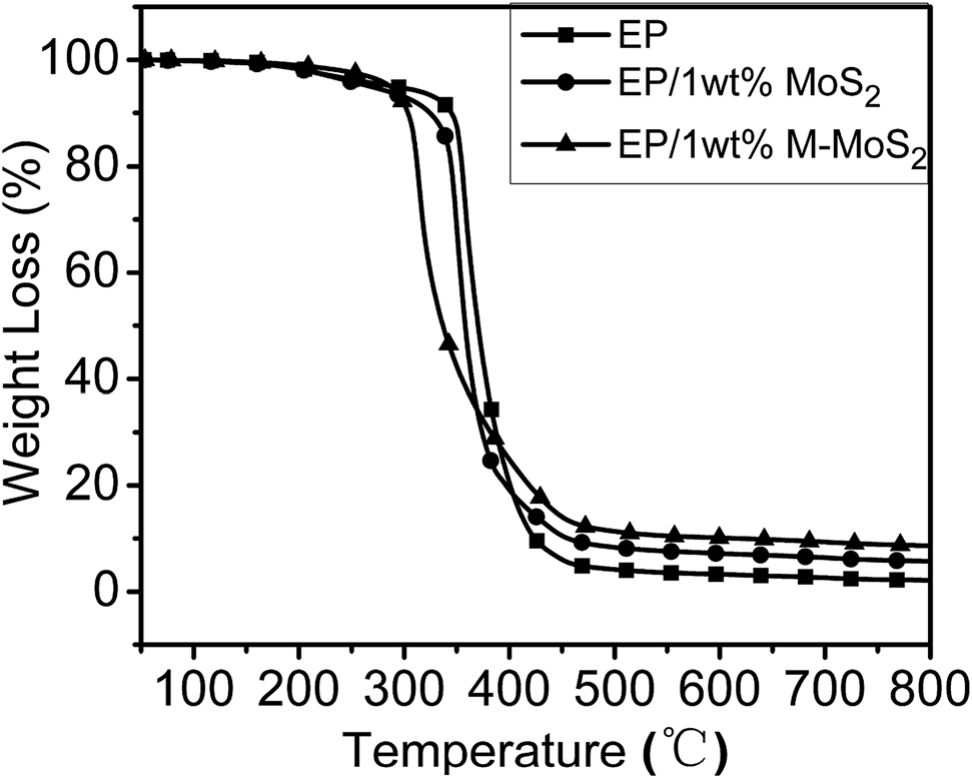
TGA curves of EP, EP/MoS_2_ and EP/M-MoS_2_ nanocomposites.

Thermal stabilities of the EP, EP/MoS_2_ and EP/M-MoS_2_ were evaluated by TGA under nitrogen atmosphere. As can be observed in Fig. 6, all the nanocomposites present similar degradation behaviors, suggesting that the existence of MoS_2_ and M-MoS_2_ did not significantly affect the degradation mechanism of the matrix polymers. For the EP/MoS_2_ and EP/M-MoS_2_ nanocomposites, their degradation temperature is lower than that of pure EP, which could be attributed to the earlier thermal degradation of melamine functional groups on MoS_2_ surface and/or the high thermal conductivity of MoS_2_. However, the addition of MoS_2_ or M-MoS_2_ fillers exhibited higher char residues compared to neat EP. Additionally, it can also be seen that the weight loss rates of the EP/M-MoS_2_ nanocomposites was lower than the EP and EP/MoS_2_ nanocomposites. This phenomenon played an important role in improving the flame retardancy of the EP resins. When increasing the temperature, the melamine degraded at first and form char on the MoS_2_ surface. The formed char can provide a protective shield of mass and heat transfer, which slow down the heat release rate during the thermal degradation process.

**Fig. 7:**
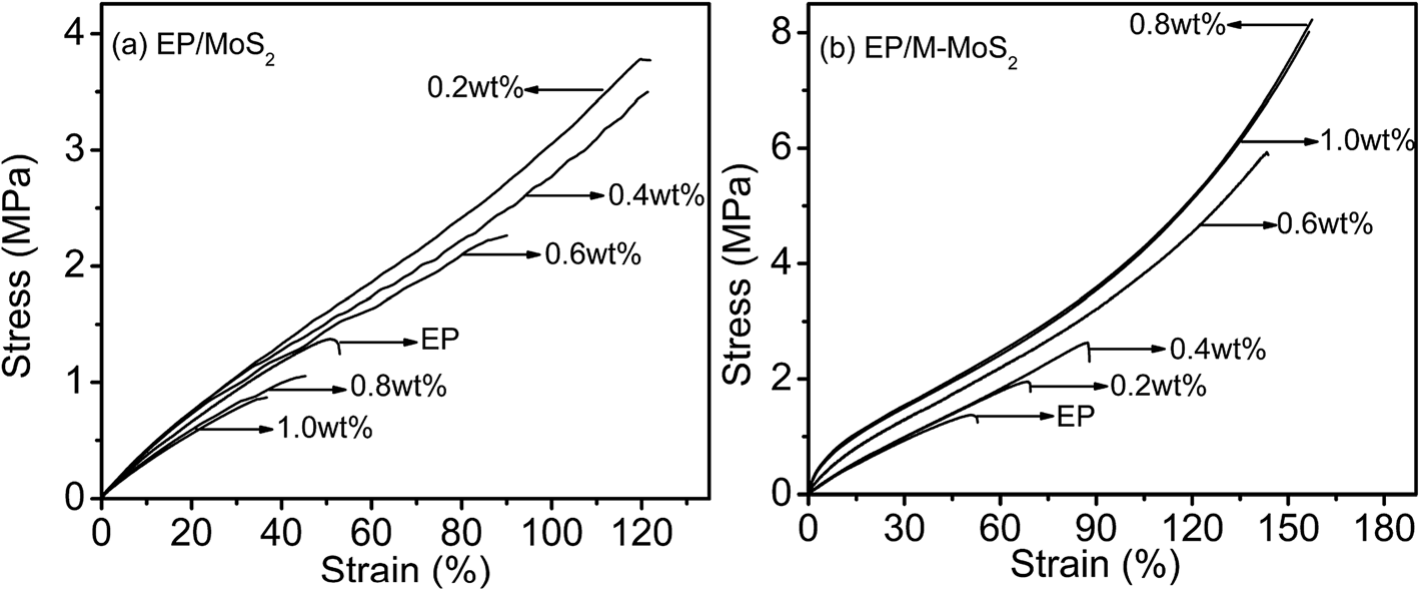
Stress–strain curves of EP, EP/MoS_2_ and EP/M-MoS_2_ nanocomposites.

**Scheme 2:**
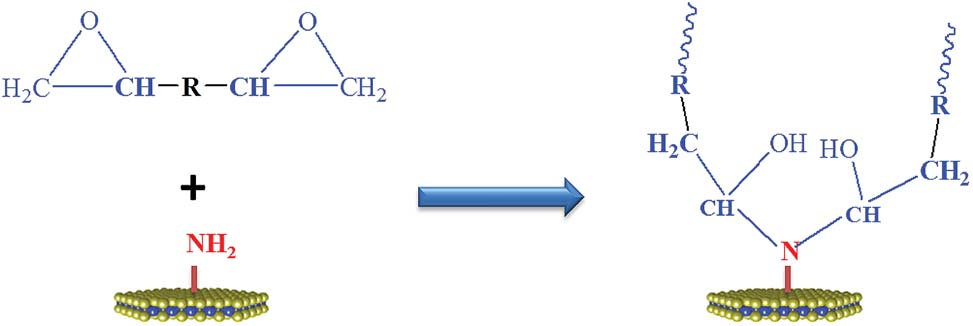
Reaction between EP and M-MoS_2_.

The influence of MoS_2_ and M-MoS_2_ on the mechanical properties of the EP nanocomposite is evaluated using a Universal Testing Machine (UTM). The reinforcing effects of the MoS_2_ and M-MoS_2_ on the tensile properties of the EP composites are summarized in Fig. 7 and Table 1. Clearly, the tensile strength and modulus of the resultant EP/M-MoS_2_ nanocomposites were significantly enhanced, even at very low M-MoS_2_ nanofiller loadings. The tensile modulus of EP/M-MoS_2_ nanocomposites increased from 3.7 to 18.6 MPa (approximately a 400% increase over neat EP), and the tensile strength increased from 1.5 to 8.3 MPa (approximately a 450% increase over neat EP) when the M-MoS_2_ content increased from 0 to 0.8 wt%. When the M-MoS_2_ content higher than 0.8 wt%, the tensile strength and modulus value barely changed. Upon introduction of MoS_2_, as the MoS_2_ content increased from 0 to 0.4 wt%, the tensile strength and modulus of EP/MoS_2_ nanocomposites slightly increased, but a reducing trend was observed for the further increasing MoS_2_ content. This phenomenon could be attributed to the agglomerate of MoS_2_ fillers and reduced the effective contact area between the MoS_2_ surface and the EP matrix, and thus reduced the reinforcement efficiency. At the same time, melamine as a modifier not only improves the interfacial compatibility between molybdenum disulfide and EP, but also acts as a co-curing agent to promote cross-linking of EP (Scheme 2). Therefore, the addition of M-MoS_2_ greatly improves the mechanical properties of EP.

**Table 1:** Mechanical properties of EP, EP/MoS_2_ and EP/M-MoS_2_ nanocomposites

	Filler content (wt%)	Tensile strength (MPa)	Tensile modulus (MPa)	Elongation at break (%)
MoS_2_	0	1.5 ± 0.2	3.7 ± 0.2	58.2 ± 5.5
	0.2	3.8 ± 0.2	3.8 ± 0.1	123.3 ± 5.8
	0.4	3.7 ± 0.4	4.6 ± 0.7	123.3 ± 5.8
	0.6	2.2 ± 0.1	4.2 ± 0.4	88.5 ± 3.5
	0.8	0.9 ± 0.1	3.2 ± 0.2	43.7 ± 2.6
	1.0	0.9 ± 0.1	3.2 ± 0.1	36.4 ± 1.4
M-MoS_2_	0	1.5 ± 0.2	3.7 ± 0.2	58.3 ± 5.5
	0.2	2.1 ± 0.2	4.0 ± 0.1	72.0 ± 4.2
	0.4	2.6 ± 0.2	4.1 ± 0.3	88.0 ± 5.0
	0.6	6.2 ± 0.3	10.5 ± 2.7	143.3 ± 5.8
	0.8	8.3 ± 0.1	18.6 ± 1.2	160.0 ± 0.0
	1.0	7.9 ± 0.3	19.7 ± 4.3	153.3 ± 11.5

## Conclusions

In summary, M-MoS_2_ fillers were successfully prepared through exfoliation of MoS_2_ followed by reaction with melamine. The effects of M-MoS_2_ on the thermal and mechanical properties of EP were investigated. Because the good dispersion and strong interfacial adhesion of M-MoS_2_ fillers and the EP matrix, the mechanical properties of EP were significantly improved, even with very low M-MoS_2_ addition. Therefore, this work provides a facile way to produce of high-performance EP nanocomposites.

## Conflicts of interest

There are no conflicts to declare.

## Supplementary Material

RA-008-C8RA02689K-s001
