# Supplementary material for: Mechanical properties of epoxy nanocomposites filled with melamine functionalized molybdenum disulfide
Source: RSC Adv. 2018 Jun 5;8(36):20450–5. doi: 10.1039/c8ra02689k (PMC9080842; doi:10.1039/c8ra02689k)
Supplement: RA-008-C8RA02689K-s001 [file RA-008-C8RA02689K-s001.pdf]

Electronic Supplementary Material (ESI) for RSC Advances.
This journal is © The Royal Society of Chemistry 2018

# Mechanical properties of epoxy nanocomposites filled with melamine functionalized molybdenum disulfide

Bin Chen[a], Bao-Jian Ni[a], Wen-Tao Liu[b], Qiu-Yang Ye[b], Si-Yuan Liu[b], He-Xin Zhang[b,c*], Keun-Byoung Yoon[c*]

a. School of Materials Science and Engineering, Shenyang University of Chemical Technology, Shenyang 110142, China

b. School of Chemistry & Chemical Engineering, Anhui University of Technology, China

c. Department of Polymer Science and Engineering, Kyungpook National University, South Korea

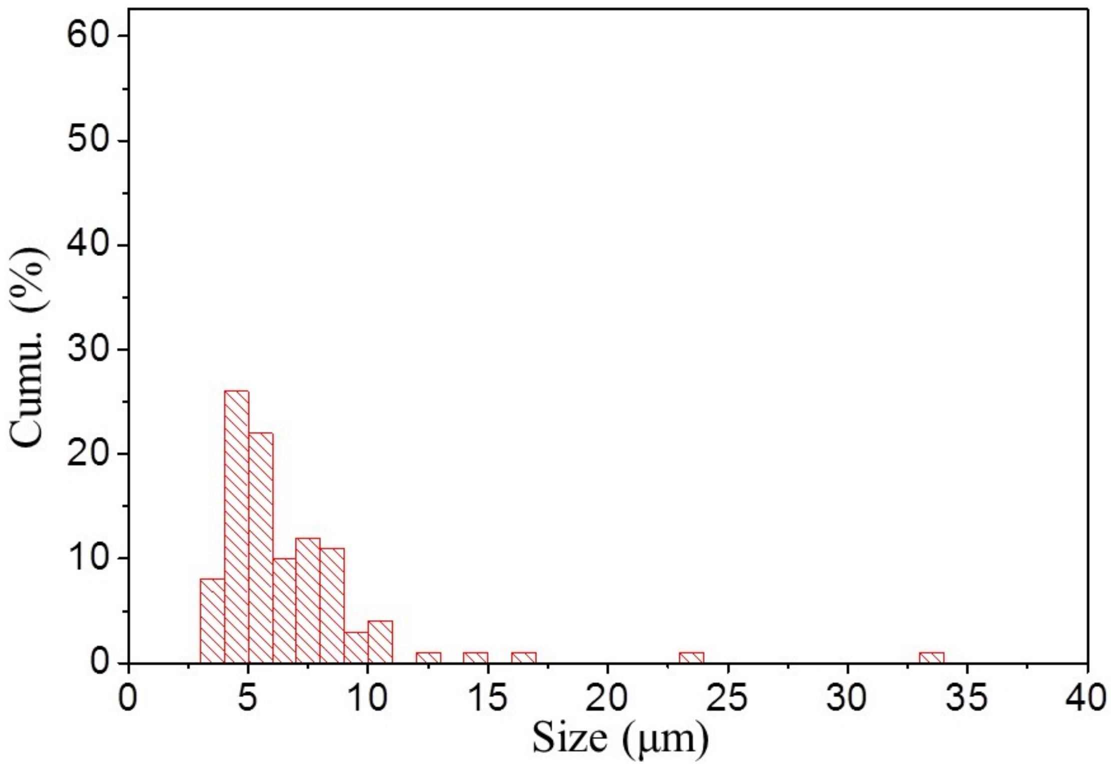


Figure S1. Size distribution of $MoS_2$

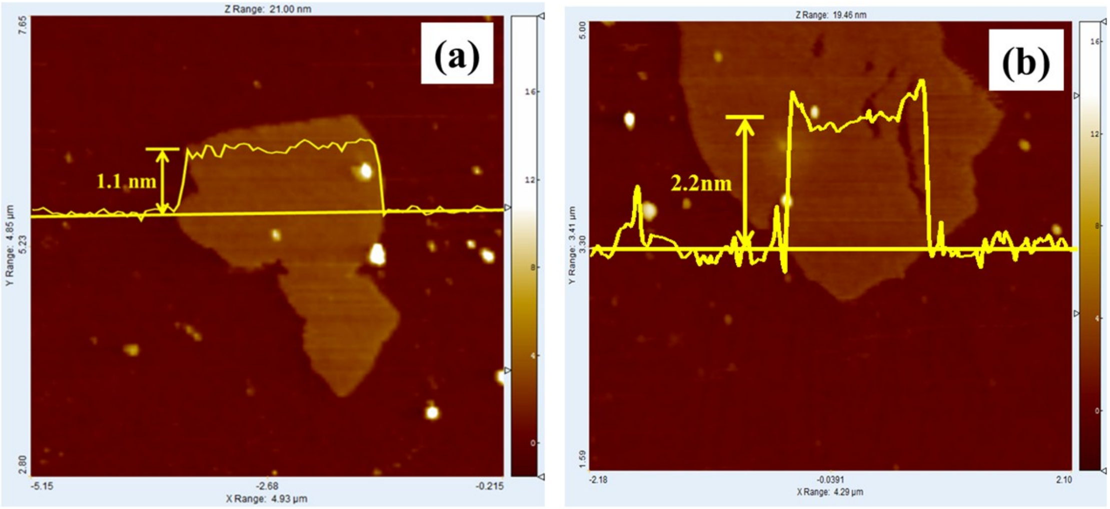


Figure S2. AFM image of exfoliated $MoS_2$ and M-$MoS_2$

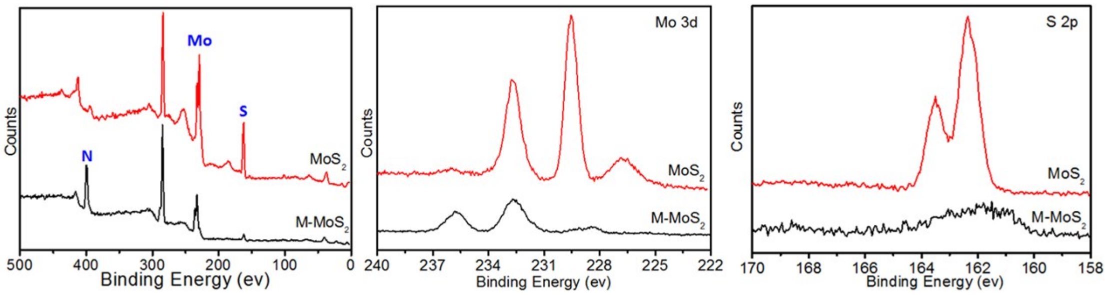


Figure S3. XPS curves of $MoS_2$ and M-$MoS_2$

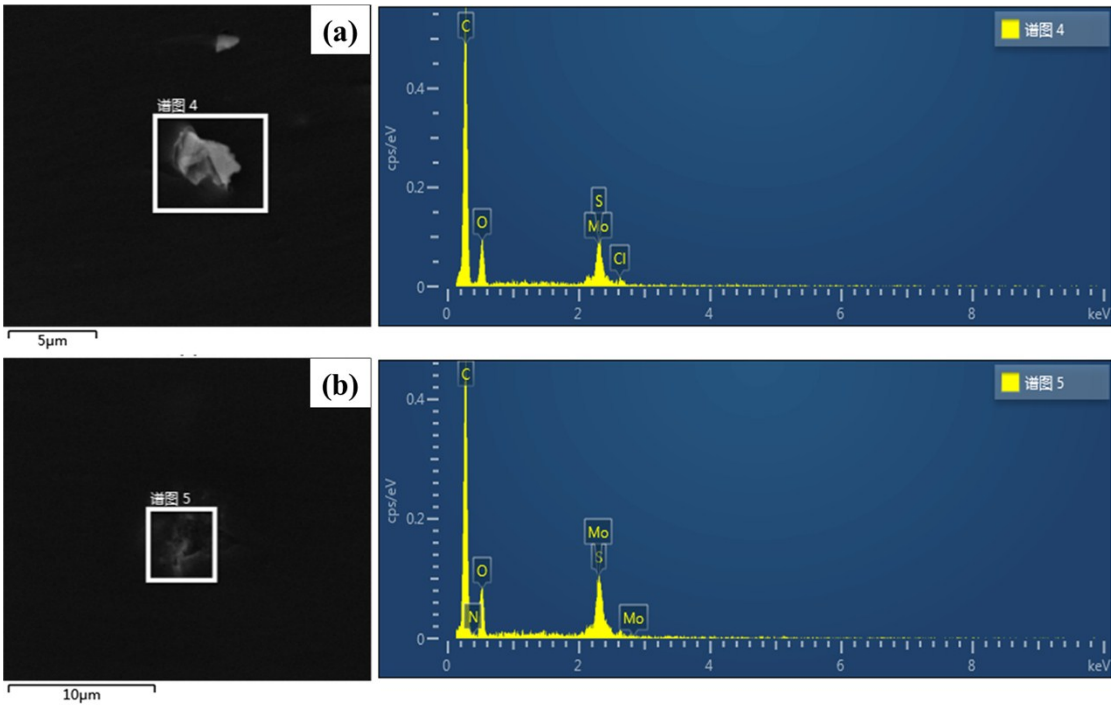


Figure S4. EDS analysis of (a)EP/$MoS_2$ and (b) EP/M-$MoS_2$ nanocomposite with 1 wt% filler addition
